# Chemical Composition, Antibacterial Properties and Mechanism of Action of Essential Oil from Clove Buds against *Staphylococcus aureus*

**DOI:** 10.3390/molecules21091194

**Published:** 2016-09-08

**Authors:** Jian-Guo Xu, Ting Liu, Qing-Ping Hu, Xin-Ming Cao

**Affiliations:** 1 College of Food Sciences, Shanxi Normal University, Linfen 041004, China; sxsd456@sina.com; 2 College of Life Sciences, Shanxi Normal University, Linfen 041004, China; liuyu1961119@163.com (T.L.); hqp72@163.com (Q.-P.H.)

**Keywords:** essential oil, *Staphylococcus aureus*, alkaline phosphatase, membrane permeability, electron microscope, protein, DNA

## Abstract

The essential oil of clove has a wide range of pharmacological and biological activities and is widely used in the medicine, fragrance and flavoring industries. In this work, 22 components of the essential oil obtained from clove buds were identified. Eugenol was the major component (76.23%). The essential oil exhibited strong antibacterial activity against *Staphylococcus aureus* ATCC 25923 with a minimum inhibitory concentration (MIC) of 0.625 mg/mL, and the antibacterial effects depended on its concentration and action time. Kill-time assays also confirmed the essential oil had a significant effect on the growth rate of surviving *S. aureus*. We hypothesized that the essential oil may interact with the cell wall and membrane first. On the one hand it destroys cell wall and membranes, next causing the losses of vital intracellular materials, which finally result in the bacterial death. Besides, essential oil penetrates to the cytoplasmic membrane or enters inside the cell after destruction of cell structure, and then inhibits the normal synthesis of DNA and proteins that are required for bacterial growth. These results suggested that the effects of the clove essential oil on the growth inhibition of *S. aureus* may be at the molecular level rather than only physical damage.

## 1. Introduction

Food poisoning and spoilage caused by microorganisms results in enormous losses of foods, and has been of vital concern to public health [1]. Many synthetic preservatives are used to control microbial growth in foods resulting in reduced incidences of foodborne illnesses and an extension in the shelf-life of food products [2,3], however, their use is being questioned due to perceived possible health concerns and potential increases in microbial resistance [4]. Therefore, the development and utilization of natural, safe and effective antimicrobial agents are desired [4,5].

Most natural plant-derived antimicrobials can be highly effective in for controlling foodborne pathogenic bacteria and extending shelf-life [6]. In addition, as in most of the cases, plants or their extracts are believed to be relatively safer for humans [2,7]. In particular, many essential oils from various plants including edible and medicinal plants, herbs, and spices have been reported to be safe and possess strong antimicrobial effects [8,9,10], and they have obtained the application in medicine and food industries [1].

The essential oil extracted from the dried flower buds of clove, is used as a topical application to relieve pain and to promote healing and also finds use in medicine, fragrance and flavouring industries [11]. Clove oil has been listed as a “Generally Regarded As Safe” substance by the United States Food and Drug Administration [12]. Clove essential oil exhibits a wide range of pharmacological and biological activities such as antioxidant [13,14], antifungicidal [15], anticarcinogenic, anesthetic, and antiprotozoal effects [11,16]. Besides, some studies have reported antibacterial activity of essential oil from clove buds against several food-borne pathogens [17,18,19]. However, to the best of our knowledge, although the in vitro antimicrobial activity of clove essential oils has been reported earlier, very little is known about its antibacterial mechanism of action. *S. aureus* is well-known for being resistant towards some antibiotics and for its production of several enterotoxins that cause many enteritis types and septicaemia [20]. Therefore, *S. aureus* was selected as the model organism to evaluate the antibacterial properties and mode of action of the essential oil from clove buds.

## 2. Results

### 2.1. Chemical Compositions of Essential Oil

The essential oil was obtained as a light yellow transparent liquid and had specific clove aroma with a yield of 12.8% (*v*/*w*). The chemical composition of essential oil was analyzed by GC and GC-MS, and the result was presented (Table 1). In total, 22 components in essential oil were identified, representing 95.80% of the total amount. The eugenol (76.23%) was found to be the major component of the essential oil, followed by *β*-caryophyllene (11.54%), caryophyllene oxide (4.29%), and eugenyl acetate (1.76%).

### 2.2. DIZ and MIC of Essential Oil

The essential oil from clove buds had a satisfactory antibacterial activity on *S. aureus* (Figure S1). The DIZ values were 16.5 mm and 20.4 mm when the concentration of essential oil was 25% and 50%. The MIC value of essential oil was 0.625 mg/mL (Figure S2), entirely inhibiting the normal growth of tested *S. aureus*. Under the same experimental conditions, the DIZ values of eugenol were 17.1 mm and 21.2 mm and its MIC value was 0.625 mg/mL, while the DIZ values of β-carophyllene were 12.3 mm and 15.8 mm and its MIC value was 1.25 mg/mL, which indicated that the antibacterial activity of essential oil from clove buds on *S. aureus* was mainly attributed to eugenol as the major component. Meanwhile, the results also implied that the antibacterial effect of the essential oil from clove buds depended on its dose.

### 2.3. Kill-Time Analysis

**Figure 1:**
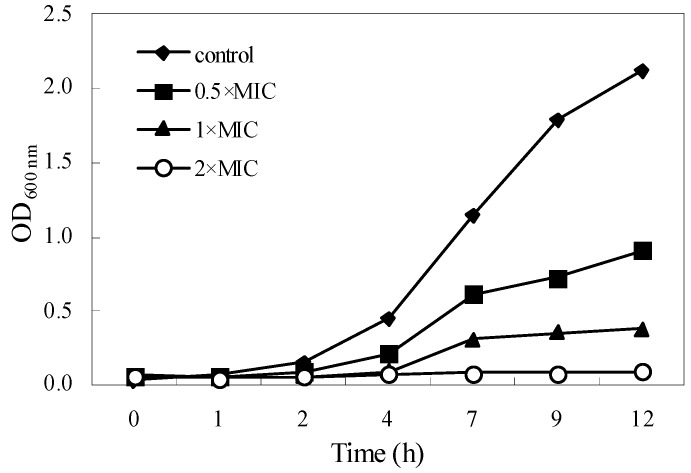
Effect of the essential oil on the viability of *S. aureus.*

The optical density (OD) values of the control and treatments at 600 nm had no obvious change within 2 h after treating (Figure 1). Thereafter, the OD values of control significantly and rapidly increased, indicating that *S. aureus* entered upon logarithmic phase. Compared to the control, *S. aureus* treated with the essential oil at the 0.5 × and 1 × MIC showed a slow increase in the trend of OD values during 12 h of incubation, but OD values were far below the control; while the OD values of treatment at 2 × MIC had on change during 12 h of incubation. These results confirmed the antibacterial activity of essential oil and showed a significant effect on the growth rate of surviving *S. aureus*, supporting the results stated above, and showed that the treatment time and concentration of the essential oil had great influences on antibacterial effects.

### 2.4. Cell Permeabilization Assay

**Figure 2:**
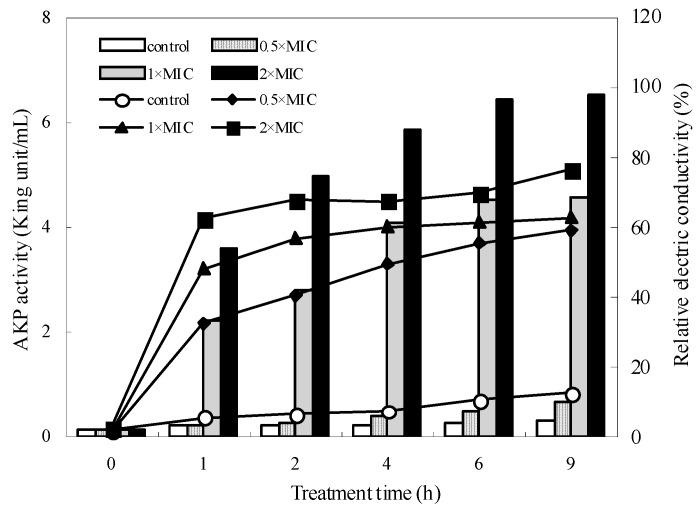
Effect of the essential oil on the AKP activity (bar) and cell membrane impermeability (line) of *S. aureus.*

The alkaline phosphatase (AKP) is located between cell wall and cell membrane and could not leak to the outside of the bacteria, hence one cannot measure its activity under normal conditions, but, when the cell wall is damaged, AKP will leak outside the bacteria. As shown in Figure 2, the AKP activity of the control of *S. aureus* showed no obvious change and increased from 0.16 to 0.31 King unit/100 mL during incubation, which may be due to normal bacterial lysis and death.

When *S. aureus* suspensions were treated with different concentrations of essential oils, the AKP increased with the prolongation of the incubation time and the increase of essential oil concentration. The AKP activity increased continuously and significantly from 0.16 to 0.65, 4.58, and 6.52 King unit/100 mL after treatment with 0.5 ×, 1 ×, 2 × MIC essential oils, respectively. The increased release of AKP indicated that the essential oil can destroy the cell walls of *S. aureus* strains, resulting in the increase of the permeability of cell wall and the destruction of the cell structure.

### 2.5. Cell Membrane Permeability

Similar to changes of the AKP, a slight increase in the relative electric conductivity of the control *S. aureus* was found, which may result from normal lysis and death of bacteria (Figure 2). Compared to the control, the relative electric conductivity of the suspension increased immediately after the addition of essential oil at greater than or equal to 0.5 × MIC concentrations and it also increased rapidly with increasing treatment time and concentration of essential oil. After incubation for 9 h, the relative electric conductivity of *S. aureus* at 0.5, 1, and 2 × MIC increased from about 2% or so to 57.43%, 60.94%, and 73.75%, respectively. This meant that the permeability of bacteria membrane would be increased correspondingly, which caused the leakage of intracellular ingredient, especially losses of electrolytes including K^+^, Ca^2+^, Na^+^ and so on.

### 2.6. Integrity of Cell Membrane

Table 2 shows the results when *S. aureus* were treated with essential oils for 4 h, respectively. The results indicated that after adding the corresponding essential oil to strains, the cell constituents' release increased significantly with the increased concentration of the essential oil. Compared to control, the concentrations of proteins, reducing sugars and cell constituents (OD_260nm_) in suspensions treated with 0.5 × MIC essential oil increased by 2.98, 1.61, 4.17 times, respectively, and they increased by 3.89, 3.34, 9.65 times respectively when treatment at 1 × MIC, while they increased by 5.42, 6.06, 21.48 times respectively when treatment at 2 × MIC. These results indicated that the irreversible damage to the cytoplasmic membrane might occur, which led to the losses of cell constituents such as protein, nucleic acids, and some essential molecules.

### 2.7. Microscopic Observations

**Figure 3:**
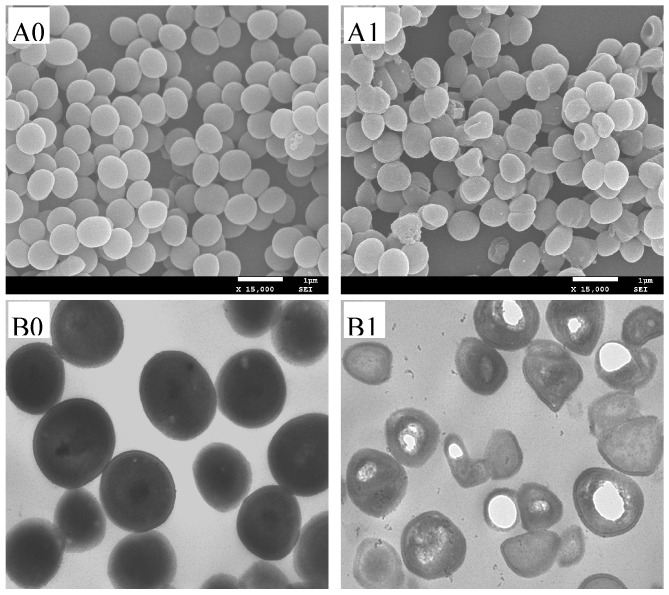
The SEM and TEM photography of *S. aureus*. **A0** and **B0**, untreated bacteria; **A1** and **B1**, bacteria treated with the essential oil at 1 × MIC.

Figure 3 showed the SEM images of the treated and untreated bacteria. The surfaces of the treated strains underwent some morphological changes compared with the untreated controls. Untreated cells were spherical, regular and showed a smooth surface (Figure 3, A0), while treated cells became irregular, pitted, and shriveled (Figure 3, A1). Figure 3 also showed the TEM image of the *S. aureus* after treatment. In micrographs of *S. aureus*, the control cell wall and membrane remained intact with uniformly distributed cytochylema (Figure 3, B0). However, after treatment with essential oil, some cells changed from their normal spherical shape into irregular, and the cell wall and cytoplasmic membrane became uneven, and some lysis was seen (Figure 3, B1). Besides, it was observed that parts of the cell wall were broken and the disruption of the outer membrane structure with membrane sloughing and breaching, which may give rise to the leaching out of cell content, which was consistent with the results of SEM and supported the results of integrity of cell membrane assays.

### 2.8. SDS-PAGE of Proteins from Bacteria

**Figure 4:**
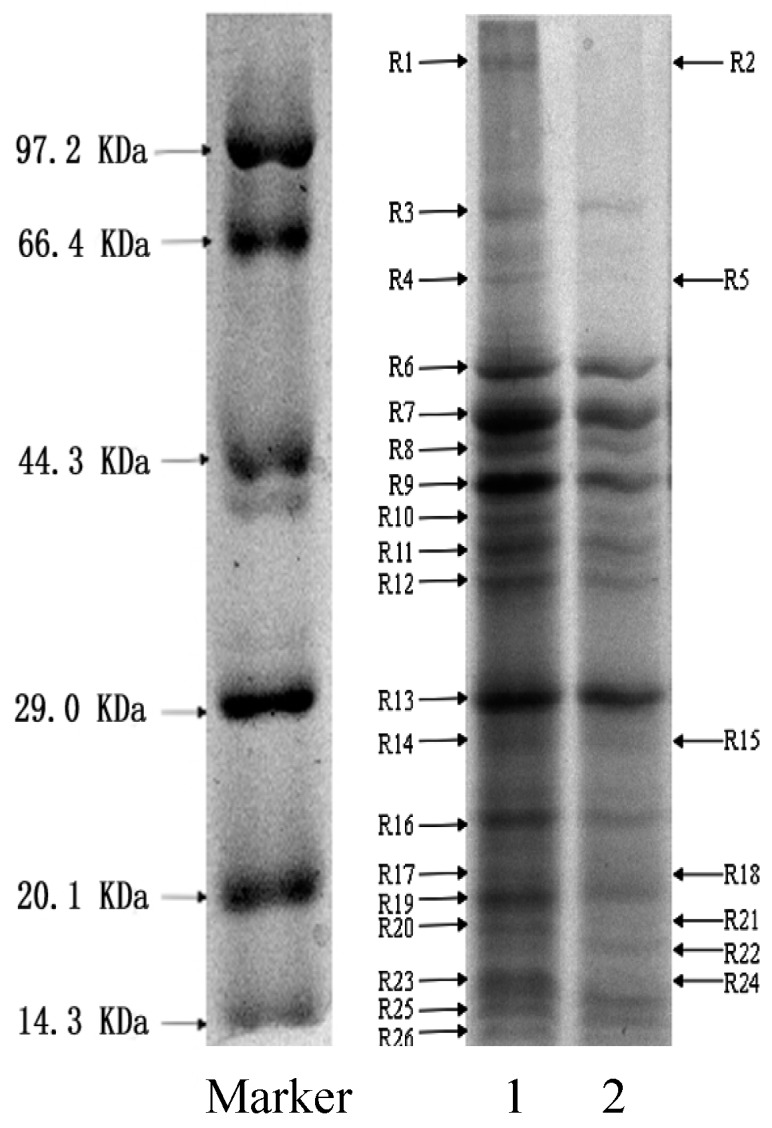
SDS-PAGE of proteins from *S. aureus*. lane 1, untreated bacteria; lane 2, bacteria treated with the essential oil at 1 × MIC.

Similarly with the protein contents leakage as above, there were higher protein contents in the supernatant than their respective controls, suggesting the permeabilizing action caused by essential oil. However, whether the essential oil has a direct action on proteins in cells needs to be further analyzed by SDS-PAGE for their soluble proteins. Protein electrophoresis bands of treated *S. aureus* were different from their controls in Figure 4.

The untreated cell protein electrophoresis bands appeared strong and clear. After treated with the essential oil at 1 × MIC for 4 h, bands of all-molecular-weight proteins became obviously shallow, and even disappeared. Considering the increased protein contents in the cell-free supernatant, it was suggested that the essential oil decreased the content of cellular soluble proteins by permeating and disrupting cell membranes. There were four thick bands (R6, R7, R9 and R13, about 50.0–40.0 and 29.0 kDa) among 19 major bands for untreated bacteria (Figure 4, lane 1). After treatment with essential oil, the amount of R15 significantly reduced, while five bands (R2, R5, R18, R21, and R24) disappeared (Figure 4, lane 2). One new band (R22, approximately 20.1 kDa) appeared in lane 2. It implied that the essential oil had a remarkable effect on bacterial proteins either by destroying them or by inhibiting their synthesis, which can result in their inactivation, and the reason of new protein bands appearance was probably due to aggregation of bacterial proteins caused by the essential oil.

### 2.9. DNA Synthesis Inhibition and DNA-Binding Assays

**Figure 5:**
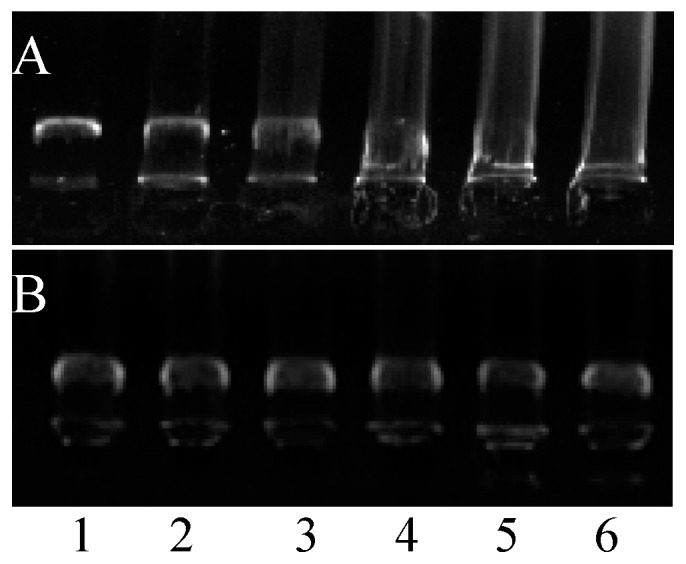
The agarose gel electrophoresis of bacterial DNA (**A**) and plasmid DNA (**B**). lane 1, the control; lanes 2–6, the samples treated with 0.25 ×, 0.5 ×, 1 ×, 2 × MIC, and 4 × MIC essential oils, respectively.

The DNA bands of control *S. aureus* were complete and clear, which means the bacteria can synthesize DNA normally (Figure 5A). However, when treated with different concentrations of essential oil from clove buds, the DNA bands were no longer clear and even disappeared in high concentration treatments, which indicated that the DNA synthesis of *S. aureus* was inhibited by essential oil of clove buds. To clarify the molecular mechanism of the essential oil, the capacity of the essential oil to bind plasmid DNA was measured by analyzing the electrophoretic mobility of DNA bands at the various concentrations of the essential oil to DNA. As shown in Figure 5B, the essential oil did not inhibit DNA electrophoretic mobility at all concentrations. The results indicated that the essential oil did not possess DNA-binding affinity.

## 3. Discussion

In this study, 22 components in essential oil from clove buds were identified and the eugenol (76.23%) was the major component of the essential oil. This is supported by previous studies [21,22,23]. However, there were differences in the content of eugenol. For example, Jirovetz et al. [21] identified 23 components of clove leaf essential oil and found that eugenol (76.8%) was the main component; Guan et al. [22] compared essential oils of clove buds extracted with supercritical carbon dioxide and other three traditional extraction methods, and found that the content of eugenol was different (50.3%–61.2%); Tang et al. [23] identified 19 components in the essential oil of clove buds and the content of eugenol was 62.21%. Although we can find in the literature studies using the essential oil from clove buds, these differences in the content and components of essential oil are difficult to compare because the species, geographic regions, and extract methods used are different among studies. More importantly, the main antibacterial components of clove oil are believed to be eugenol [24].

The results from the Oxford cup method and MIC, indicated that the essential oil from clove buds had inhibitory effects against *S. aureus*, which was supported by previous studies [18,19]. Goñi et al. [17] reported the MIC of the essential oil from clove buds against *S. aureus* was 27 mg/L; Lu et al. [18] suggested the MIC of the clove essential oil against *S. aureus* was 0.8 mg/mL; Ivanovic et al. [19] reported the MIC of the clove essential oil against *S. aureus* ATCC 25923 was 0.64 mg/mL. The differences in antibacterial effects of essential oil against *S. aureus* may be linked to experimental conditions such as inoculation amount of bacteria, incubation time, and the sources of essential oil and so on. Besides, these studies reported the inhibitory effect against other pathogens such as *Listeria monocytogenes*, *Escherichia coli* [17,18,19].

The clove essential oil is widely used in the medicine, fragrance and flavoring industries and has been listed as a “Generally Regarded As Safe” substance by the United States Food and Drug Administration [11,12]. The U.S. National Institutes of Health reported there were no significant observable differences between treated and control groups of rats for carcinogenesis, either non-neoplastic (toxic) lesions or neoplasms that could be attributed to eugenol [25]; Taher et al. [26] have reported that the median (intraperitoneal) LD_50_ of clove oil was 161.9 mg/kg, which indicates that the toxicity of the clove oil is limited. In this study, the MIC value of essential oil was 0.625 mg/mL, and 0.0625% of the essential oil in food is relatively low. Based on the above reasons, clove essential oil at the concentrations used in the antibacterial assays is likely to be safe, which also requires further research.

Although the antibacterial properties of essential oils and their components have been reviewed in the past, the mechanism of action has not been studied in great detail [2,27]. Considering the large number of different groups of chemical compounds in essential oils, it is most likely that their antibacterial activity is not attributable to one specific mechanism but that there are several targets in the cell [2]. Therefore, it is necessary to clarify fully the mode of action of essential oil from clove buds against the *S. aureus* in several respects including changes in cell shape and structure, intracellular substances such as cell critical molecules, ions, and other cell contents.

In this study, the increased release of AKP into the culture medium indicated that the essential oil can destroy the cell wall of *S. aureus* strains, resulting in the increase of the permeability of the cell wall. The swift increase of the relative electric conductivity of the treatments implied that the permeability of the membrane would be increased, which caused the leakage of intracellular ingredient, especially losses of electrolytes including K^+^, Ca^2+^, Na^+^ and so on; while losses of the reducing sugars and macromolecules including protein, nucleic acids from *S. aureus* strains further confirmed and showed the bacteria membrane was more damaged, and the irreversible damage to cell shape and structure of *S. aureus* were also confirmed by the results of SEM and TEM. Once the cell structure is destroyed, whether small molecules such as some reducing sugars, K^+^, Ca^2+^, Na^+^ or macromolecules including protein, nucleic acids released from the bacteria to the outside. Small ions such as K^+^, Na^+^, Ca^2+^ are necessary electrolytes for the maintenance of the energy status, regulation of metabolism, solute transport and so on, which can result in changes of cell membrane structure, detrimentally affect cell metabolism and lead to cell death [28]. The macromolecules including proteins, and nucleic acids which reside throughout the interior of the cell are the living matter of cells. The transfer of cellular information through the processes of translation, transcription and DNA replication occur within the same compartment and can interact with other cytoplasmic structures [8,29]. Therefore, we concluded that one of antibacterial modes of action on *S. aureus* was that the essential oil from clove buds first destroyed the cell wall and membranes, next causing the losses of intracellular materials, which finally resulted in the bacterial death.

All bacterial cells depend on the synthesis of DNA and protein for their growth. Thus, any interference with the synthesis of DNA or protein can block the growth of a bacterium [30,31]. The SDS-PAGE results of protein implied that the essential oil had a remarkable effect on proteins of *S. aureus* either by destroying them or by inhibiting their synthesis (Figure 4). The agarose gel retardation assays revealed that the essential oil from clove buds was not capable of binding plasmid DNA (Figure 5B), suggesting that DNA may not be the major intracellular target for essential oil. However, the macromolecular synthesis assays showed the essential oil is capable of blocking synthesis of both DNA and protein in bacterial cells (Figure 3 and Figure 4). This demonstrated that the essential oil of clove buds might exert an inhibitory action during the processes of transcribing the DNA strand into mRNA, the translation of the mRNA into protein and DNA replication itself, or one or two of them. Therefore, we concluded that another mechanism of antibacterial action of essential oil from clove buds against *S. aureus* was by inhibition of bacterial DNA and protein synthesis. Although this work clearly indicated that the essential oil from clove buds can inhibit DNA and protein synthesis, further work is necessary and is in progress to determine in detail how it acts on the cell. 

In this study, we just investigated the antibacterial effect and action mechanism of essential oil from clove buds against *S. aureus* in the microbial culture system, further research on the antibacterial of essential incorporated in food is still necessary since biological performance may depend on several factors including the interference among the chemicals composition of the food, the active ingredients and their bio-availability, and environment conditions such as pH, temperature, humidity. In addition, whether effective concentrations of clove oil would affect the aroma and taste of the food treated, and whether these concentrations of clove oil to be cytotoxic to eukaryotic cells, also need further study. In addition to food preservation, clove essential oil can be used as an antibacterial agent and coatings on medically-relevant surfaces or even for fabrication of functional biodegradable materials according to the present and previous research results [32,33].

## 4. Experimental Section

### 4.1. Plant Materials, Microbial Strain and Culture

Clove buds, which came from the variety *Syringa yunnanensis* Franch grown in Nanning City of Guangxi Province, were used in the study. They were obtained as a commercial product from the local market in 2014. *Staphylococcus aureus* ATCC 25923 was provided by the College of Life Science, Shanxi Normal University. The strain was cultured at 37 °C on nutrient agar (NA) or nutrient broth (NB) media.

### 4.2. Extraction of Essential Oil

The dried clove buds were ground and hydrodistilled for 4 h using a Clevenger-type apparatus. The oily layer obtained on top of the aqueous distillate was separated and dried with anhydrous sodium sulfate. The oil obtained was stored in tightly closed dark vials at 4 °C until further analysis.

### 4.3. GC-MS Analysis

The analysis of the essential oil were performed using a Hewlett-Packard 5890 II GC (Hewlett-Packard, Palo Alto, CA, USA), equipped with a HP-5 MS capillary column (30 m × 0.25 mm; film thickness, 25 μm) and a HP 5972 mass selective detector for the separation. The mass selective detector was operated in electron-impact ionization (EI) mode with a mass scan range from *m/z* 30 to 500 at 70 eV. Helium was the carrier gas at a flow rate of 1 mL/min. A sample of 0.1 μL of the essential oil was injected manually using a 1:50 split ratio. Injector and MS transfer line temperatures were set at 250 and 200 °C, respectively. The oven temperature was programmed from 40 °C for 2 min, raised to 180 °C at a rate of 3 °C/min, held isothermal for 2 min, and finally raised to 250 °C for 5 min. The components were identified by comparing their GC retention indices, NIST mass spectral search program, and mass spectra with publish data.

### 4.4. Antibacterial Activity

The essential oil was dissolved in ethanol and sterilized by filtration through 0.22 μm Millipore filters. The antimicrobial tests were carried out by the Oxford cup method [34] using 100 μL of standardized inoculum containing 1 × 10^7^ colony-forming units (CFU)/mL of bacteria suspension poured and uniformly spread on NA. Oxford cups were placed on the inoculated agar, and then 100 μL of essential oil was added with a micropipette. The diameter of inhibition zone (DIZ) was measured after 24 h of incubation at 37 °C, and ethanol was used as negative control.

### 4.5. Minimum Inhibitory Concentration (MIC)

The MIC was determined according to the method described by Sarker et al. [35] with minor modifications. Briefly, stock solution of essential oil was prepared in ethanol, and the resazurin solution was prepared at 1 mg/mL in distilled water, sterilized by filtration, and stored at 4 °C for up to 1 week. Briefly, 240 μL of NB was dispensed in each well of a sterile flat-bottom 96-well plate and 5 μL of serial two-fold dilutions of each sample were prepared directly in the plate. The 2.5 μL of broth inoculum containing approximately 1 × 10^7^ CFU/mL microorganisms and 2.5 μL of resazurin solution was added to each well. After 24 h of incubation, a change in color from blue to pink indicated the growth of bacteria, and the MIC was defined as the lowest concentration of the sample that inhibited this change in color.

### 4.6. Kill-Time Analysis

The assay was performed according to the method described by Muroi and Kubo [36] with some modifications. The effects of the essential oil with different concentrations (0.5 ×, 1 × and 2 × MIC) on the growth of tested bacteria were studied. The cultures were incubated in NB at 37 °C and 120 rpm. At selected time intervals, samples from test culture were taken and the absorbance at 600 nm was measured.

### 4.7. Cell Wall Permeabilization Assay

Cell wall permeabilization was determined by measuring the release of alkaline phosphatase (AKP) activity from *S. aureus* into the culture medium. After incubated at 37 °C for 10 h (1 × 10^7^ CFU/mL), the essential oils at different concentrations (0.5 ×, 1 × and 2 × MIC) were added into bacteria solution. After completely mixed, the cultures were incubated at 37 °C and shaken with a rotary shaker (Shanghai Shengke Instruments, Shanghai, China) at 120 rpm. Samples from test culture were taken at 1, 2, 4, 6 and 9 h during incubating process and centrifuged at 5000 rpm for 10 min. The AKP activity in supernatant was examined using an AKP kit according to the method described by manufacturer (Nanjing Jiancheng Bioengineering Institute, Nanjing, China).

### 4.8. Permeability of Cell Membrane

The permeability of bacteria membrane is expressed in the relative electric conductivity and determined as previously reported [9]. After incubated at 37 °C for 10 h, *S. aureus* strains were separated by centrifugation (Eppendorf Ltd., Hamburg, Germany) at 5000 rpm for 10 min. Then the bacteria were washed with 5% of glucose until their electric conductivities were near to that of 5% glucose. The samples at different concentrations were added to 5% glucose and the electric conductivities were marked as L_1_. Then different concentrations of samples were added into the isotonic bacteria solution. After complete mixing, the samples were incubated at 37 °C for 8 h, and then the conductivities were measured and marked as L_2_. The conductivity of bacteria in 5% glucose treated in boiling water for 5 min was served as the control and marked as L_0_. The permeability of bacteria membrane is calculated according to the formula:

Relative electric conductivity (%) = 100 × (L_2_ − L_1_)/L_0_. (1)

### 4.9. Integrity of Cell Membrane

The cell integrity of *S. aureus* strains is examined by determining the release of cell constituents into supernatant according to the method previously reported [9]. Cells from the 100 mL working culture of *S. aureus* were collected by centrifuged for 15 min at 5000 rpm, washed three times, and resuspended in 0.1 M phosphate buffer solution. One hundred milliliters of cell suspension were incubated at 37 °C under agitation for 4 h in the presence of sample at different concentrations. Then, 25 mL of samples were collected and centrifuged at 11,000× *g* for 5 min. And then the concentrations of proteins and reducing sugars in supernatant were determined. In addition, to determine the concentration of the released constituents consisting largely of nucleic acids, 3 mL supernatant was used to measure UV absorption at 260 nm. 

### 4.10. Scanning Electron Microscope (SEM) and Transmission Electron Microscope (TEM)

The treatment of bacterial cells and observations of SEM and TEM were respectively performed according to the method described by Diao et al. [37]. The cells were incubated in NB at 37 °C for 10 h, and then the suspensions were added 1 × MIC of sample. The suspension was incubated at 37 °C for 4 h, and then the suspension was centrifuged at 5000 rpm for 10 min. The cells were washed twice with 0.1 M PBS (pH 7.4) and fixed with 2.5% (*v*/*v*) glutaraldehyde in 0.1 M PBS overnight at 4 °C. After this, half of the cells were successively dehydrated using 30%, 50%, 70%, 90%, and 100% ethanol, and then the ethanol was replaced by tertiary butyl alcohol. Then, cells were gold-covered by cathodic spraying, and morphology of the bacterial cells was observed on a scanning electronic microscope (SEM, JSM-7500F, JEOL Ltd., Tokyo, Japan) operated at an accelerating voltage of 25–30 kV. The other half of bacteria cells were postfixed with 1% (*w*/*v*) OsO_4_ in 0.1 M PBS for 2 h at room temperature and washed three times with the same buffer, then dehydrated by a graded series of ethanol solutions (30%, 50%, 70%, 90%, and 100%). Stained bacteria were viewed and photographed with a transmission electronic microscope (H-600, Hitachi Ltd., Tokyo, Japan) operated at 75 kV.

### 4.11. Sodium Dodecyl Sulphate Polyacrylamide Gel Electrophoresis (SDS-PAGE)

The assay was performed according to the method described by Sánchez et al. [38] with some modifications. Cells of *S. aureus* were prepared and treated as described above. The suspensions were incubated at 37 °C for 4 h, and then the treated cells were collected by centrifuged for 10 min at 5000 rpm, and resuspended in 0.1 M phosphate buffer solution (pH 7.2). The final cell suspension was adjusted to an absorbance the same as the control. Equal amounts of cell suspensions were withdrawn and then centrifuged. The pellets were washed twice, resuspended in sample dilution buffer including 0.06 M Tris-HCL (pH 6.8), 5% *β*-mercaptoethanol, 10% glycerol, 2% SDS and 0.001% bromophenol blue, and heated in boiling water for 10 min. The SDS-PAGE was performed with a 5% stacking gel and a 12% separating gel followed by Coomassie Brilliant Blue staining.

### 4.12. DNA Synthesis Inhibition

The inhibition of DNA synthesis in *S. aureus* was determined as in a previous study with minor modifications [39]. The bacterial cells were incubated for 10 h in nutrient broth at 37 °C and then the suspension was added the essential oils at different concentrations (0.25 ×, 0.5 ×, 1 ×, 2 ×, and 4 × MIC). All suspensions were incubated at 37 °C for 4 h, and then centrifuged. The cells were washed twice with 0.1 M phosphate buffered solution (pH 7.4). The whole-cell DNA was collected using a rapid bacterial genomic DNA isolation kit according to the manufacturer’s instructions. Then the samples were electrophoresed on 0.8% agarose gel containing 0.5 μg/mL ethidium bromide and the DNA was photographed under ultraviolet light.

### 4.13. DNA-binding Assay

To evaluate the DNA-binding capabilities of essential oil, an electrophoretic mobility shift assay was performed according to the method described by Zhang et al. [40]. The plasmid DNA pBR322 (100 ng, in 10 mM Tris, 1 mM EDTA buffer, pH 8.0) was mixed with different concentrations of essential oil for 30 min at 37 °C. After adding 1 μL of loading buffer (10% ficoll 400, 10 mM Tris–HCl, 50 mM EDTA, 0.25% Bromophenol Blue at pH 7.5), complexes of DNA and sample were resolved by electrophoresis on a 0.8% agarose gel in Tris acetate EDTA buffer (40 mM Tris acetate and 1 mM EDTA, pH 8.0) and the migrated DNA was visualized by fluorescence of ethidium bromide, which was applied after electrophoresis.

### 4.14. Statistical Analysis

One-way analysis of variance (ANOVA) and Duncan’s tests were carried out to test significant differences (*p* < 0.05) among the treatments using Data Processing System software (DPS, version 7.05).

## 5. Conclusions

In conclusion, eugenol (76.23%) was the major component of the essential oil obtained from clove buds. The essential oil exhibited antibacterial activity against *S. aureus*, and the antibacterial effects depended on its concentration and time of action. We hypothesize that the essential oil may interact with the cell wall and membrane first. On the one hand it destroyed cell wall and membranes, next causing the losses of vital intracellular materials, which finally resulted in the bacterial death. On the other hand, it penetrated to the cytoplasmic membrane or entered inside the cell after destruction of the cell structure and then inhibited the normal synthesis of DNA and proteins that are required for bacterial growth. These results suggested that the effects of the essential oil from clove buds on the growth inhibition of *S. aureus* may be at the molecular level rather than only physical damage. However, further research on the mechanisms of action and the effect on other food spoilage and poisoning bacteria are still necessary to fully evaluate the potential of the essential oil of clove buds in foods.

## Figures and Tables

**Table 1:** Chemical composition of essential oil from clove buds.

Compound	Peak Area (%) ^a^	Compound	Peak Area (%) ^a^
2-Pinene	0.02	Eugenol	76.23
*α*-Pinene	0.03	*β*-Caryophyllene	11.54
Eucalyptol	0.14	*α*-Caryophyllene	0.64
Methyl salicylate	0.06	(−)-b-Cadinene	0.12
Chavicol	0.09	*β*-Selinene	0.25
4-Allylanisole	0.13	*α*-Selinene	0.16
Anethol	0.11	Caryophyllene oxide	4.29
*α*-Muurolene	0.01	Jasmone	0.07
*α*-Copaene	0.05	Ledol	0.03
Valencene	0.01	Globulol	0.04
Eugenyl acetate	1.76	Cedrene	0.02

^a^ Peak area obtained by GC-FID.

**Table 2:** Effects of the essential oil on cell constituents' release of *S. aureus.*

Concentrations		Cell Constituents’ Release	
	Protein (µg/mL)	Reducing Sugar (µg/mL)	Cell Constituents (OD_260nm_)
Control	12.2 ± 1.2 d	12.5 ± 1.4 d	0.023 ± 0.006 d
0.5 × MIC	48.86 ± 3.3 c	32.6 ± 2.6 c	0.119 ± 0.022 c
1 × MIC	59.75 ± 4.8 b	54.3 ± 5.1 b	0.245 ± 0.052 b
2 × MIC	78.63 ± 6.1 a	88.2 ± 4.5 a	0.517 ± 0.063 a

Values represent means of three independent replicates ± SD. Different letters within a column indicate statistically significant differences between the means (*p* < 0.05).

## Supplementary Materials

Supplementary materials can be accessed at: http://www.mdpi.com/1420-3049/21/9/1194/s1.
