# Supplementary material for: Chemical Composition, Antibacterial Properties and Mechanism of Action of Essential Oil from Clove Buds against Staphylococcus aureus
Source: Molecules. 2016 Sep 8;21(9):1194. doi: 10.3390/molecules21091194 (PMC6274078; doi:10.3390/molecules21091194)
Supplement: Supplementary file 1 [file molecules-21-01194-s001.pdf]

Supplementary Materials: Chemical Composition, Antibacterial Properties and Mechanism of Action of Essential Oil from Clove Buds against *Staphylococcus aureus*

Jian-Guo Xu, Ting Liu, Qing-Ping Hu and Xin-Ming Cao

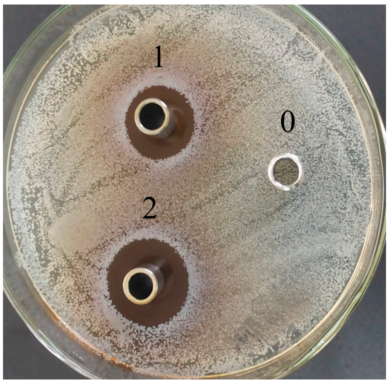

Figure S1. Effects of different concentrations of essential oil on *S. aureus*. Cup 0, control; cup 1, 12.5% essential oil; cup 2, 25% essential oil.

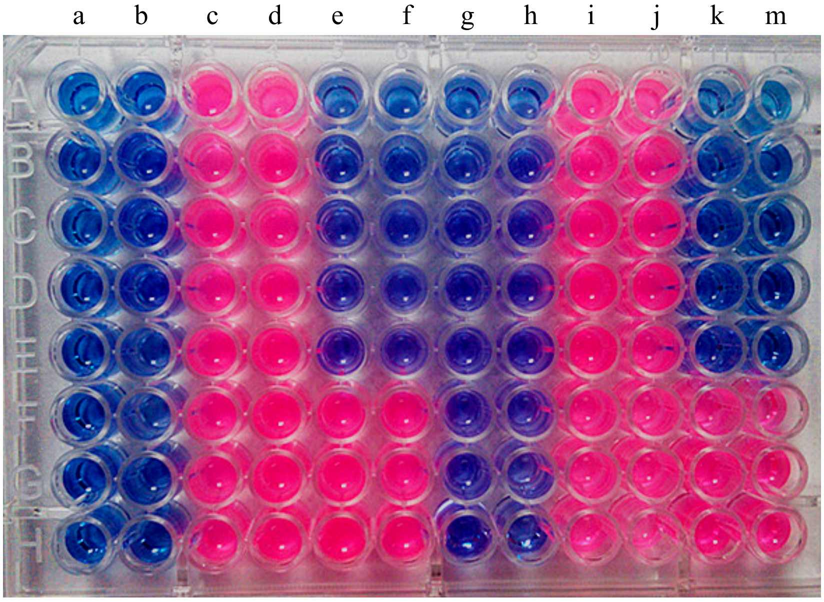

Figure S2. Plates after 24 h in modified resazurin assay (pink colour indicates growth and blue means inhibition of growth). column a, sterility control (broth + indicator), no bacteria; column b, sterility control (test compound in serial dilution + broth + indicator), no bacteria; columns c and d, control without drug (bacteria + broth + indicator); columns e and f, test essential oil (in serial dilution in wells A–H + broth + indicator + bacteria); columns g–m, replicates of columns a–f, respectively; lines A–H, treatment with 0.39%–50% essential oil, respectively.
